# Patterns of Common Dermatological Conditions among Children and Adolescents in Pakistan

**DOI:** 10.3390/medicina59111905

**Published:** 2023-10-27

**Authors:** Arfa Majeed, Sammina Mahmood, Adnan Hassan Tahir, Mehmood Ahmad, Muhammad Abu Bakr Shabbir, Waqas Ahmad, Asif Iqbal, Rana Muhammad Zahid Mushtaq, Sadaf Aroosa, Hafiz Saleet Ahmed, Naeem Rasool, Wajeeha Ramish

**Affiliations:** 1 Department of Pharmacology and Toxicology, University of Veterinary and Animal Sciences, Lahore 54000, Pakistan; 2 Department of Botany, Division of Science and Technology, Bank Road Campus, University of Education, Lahore 54000, Pakistan; 3 Department of Clinical Sciences, Faculty of Veterinary and Animal Sciences, PMAS—Arid Agriculture University, Rawalpindi 46300, Pakistan; 4 Department of Pharmacology and Toxicology, Faculty of Veterinary and Animal Sciences, The Islamia University of Bahawalpur, Bahawalpur 63100, Pakistan; 5 Institute of Microbiology, University of Veterinary and Animal Sciences, Lahore 54000, Pakistan; 6 Department of Pathology, University of Veterinary and Animal Sciences, Lahore 54000, Pakistan; 7 Department of Parasitology, Riphah International University, Lahore 54000, Pakistan; 8 Division of Infection Medicine, College of Medicine, The University of Edinburgh, Edinburgh EH16 4SB, UK; 9 Department of Livestock Management, Faculty of Veterinary and Animal Sciences, The Islamia University of Bahawalpur, Bahawalpur 63100, Pakistan

**Keywords:** fungal, dermatology, infections, Pakistan

## Abstract

*Background and Objectives:* Dermatological disorders are highly prevalent among children in Pakistan. The present cross-sectional study aims to identify the spectrum of dermatological conditions among children and adolescents in Pakistan. *Materials and Methods:* A total of 582 patients (50.9% males; 49.1% females) were included in the study based on their age (5.7 ± 4.1 years), dermatological condition, and epidemiology. The youngest patient was aged ten days, whereas the eldest was seventeen. Age criteria were further stratified into three categories: infants and toddlers (≤5 years), children (≥5 to <12 years), and adolescents (≥12 to <18 years). Amongst them, the majority was from Punjab (81.6%), while the other regions included were Azad Jammu and Kashmir (14.4%), Islamabad (3.3%), and Khyber Pakhtunkhwa (0.7%). *Results:* Scabies was the highest reported skin condition with 281 (45.55%) patients, followed by 114 (19.6%) with eczema, 60 (10.3%) with dermatitis, 33 (5.7%) with tinea capitis, 17 (2.9%) with tinea corporis, 16 (2.7%) with impetigo, and 15 (2.6%) with folliculitis. Other conditions include urticaria, burns, infections, pediculosis, tinea inguinalis, tinea faciei, nappy rashes, alopecia, warts, tinea incognito, tinea cruris, and acne vulgaris. The chi-squared test showed a high prevalence of tinea corporis and acne among adolescents (12–17 years), whereas eczema, dermatitis, and impetigo were more prevalent among infants and toddlers. *Conclusions:* Pets or livestock and poor hygiene were found to be highly reported risk factors for many dermatological conditions like scabies and fungal infections. Dermatological conditions are common in younger individuals, but unfortunately, many children do not receive the desired medical assistance.

## 1. Introduction

Dermatological infections are more prevalent among children due to poor immunological defense mechanisms at an early age [1]. Pakistan’s most prevalent dermatological conditions are scabies, impetigo, dermatitis, seborrheic dermatitis, folliculitis, urticaria, and tinea infections [2]. These disorders are caused by various causative agents, including bacteria, viruses, fungi, and parasites [3]. Predisposing factors for dermatosis and skin infections in children are multifaceted [4]. Besides immunological vulnerability owing to an immature immune system, local environmental conditions, such as high humidity, diverse insect populations, and allergen exposure, contribute significantly to skin infections and dermatoses [5]. Socioeconomic status also plays a crucial role. Children from lower-income backgrounds are at a higher risk due to inadequate hygiene practices, limited access to healthcare, and challenges affording proper skincare and treatment [6].

Scabies is Pakistan’s most prevailing dermatological infection, followed by fungal infestations [7]. It is a highly communicable disease caused by an ectoparasite, affecting 300 million people yearly [8]. This disease is responsible for 50% of dermatological infections in children in Sindh, Pakistan [9]. Fungal infections are known as “hidden killers” as the death ratio due to invasive fungal infections is equal to tuberculosis and currently exceeds malaria [10]. Tinea capitis (T. capitis), tinea pedis (T. pedis), tinea faciei (T. faciei), diaper rash, oral thrush, and tinea corporis are common fungal infections in children [11].

Eczema is a vital skin condition worldwide, holding an economic burden equal to asthma. It is a chronic skin condition involving a disturbed epidermal layer and pruritic skin, often including immune-modulatory system sensitization to specific food or allergens [12]. Patients with eczema are more prone to acquiring other skin infections [13]. In 2016, researchers suggested that a lack of “filigree” is a major cause of eczema [14]. The reason for the high prevalence of eczema in children is still unknown, but family genes account for eczema in the majority of patients [15].

Dermatological infections are fairly prominent in Pakistani children due to their developing immune systems. It was also reported that scalp diseases, besides bacterial, fungal, parasitic, and viral infections, are most prevalent among children in Pakistan [16]. The children under such circumstances are mostly treated by their guardians at home and only referred to a dermatologist when the condition worsens, making diagnosis and treatment challenging. There is an information gap about the frequency and distribution of dermatological disorders in children and adolescents in Pakistan. This cross-sectional study aims to discover the frequency and distribution of dermatological disorders in children and adolescents. The study will provide a comprehensive understanding of the skin disorders spectrum among Pakistan’s children and adolescents.

## 2. Materials and Methods

A descriptive cross-sectional study was conducted over seven months, from January to July 2021. All samples were collected according to the institutional ethical committee (IEC) guidelines, Riphah International University, Lahore (Rcvets-959). In addition, written informed consent was taken from all patients, indicating that data will be used to publish internal or external reports of Riphah International University, Lahore, and research publications (Rcvets-958).

Specific criteria were set for selecting suitable patients before initiating the study. The prime factors considered were age, dermatological condition, and patient epidemiology before selecting the participants. The sample size was calculated using the previously described method [17]. The following formula was used to calculate the sample size.

$$n=\frac{Z^{2}pq}{d^{2}}$$

where *Z*^2^ = 2.576^2^, *p* is expected prevalence (30%), *q* = 1 − *p* (70%), *d* is desired absolute precision (5%). A total of 582 (convenient sampling) participants from various cities of Punjab, Azad Jammu, and Kashmir, along with Rawalpindi and Islamabad, were enrolled in the study. Selected individuals were under the age of 17 years and were suffering from a particular skin condition. The individuals were further categorized into three groups: infants and toddlers (≤5 years), children (≥5 to <12 years), and adolescents (≥12 to <18 years).

Each individual in the study underwent a thorough examination by a dermatologist who collected their medical history and conducted a comprehensive assessment to diagnose the disease. After ensuring the patient’s well-being, the physician documented the medical history and recommended treatment on a prescription. The patient was then referred to a pharmacist for additional guidance. Before interviewing for the study, the pharmacist confirmed the patient’s current health status and obtained verbal consent from the patient and their guardian. Since the participants were children, verbal interviews were preferred over written questionnaires. Only those patients who agreed to participate in the interviews and provided complete information about their medical history were included in the study.

Recruited children and their parents were interviewed and examined in daylight. The initial diagnosis was made on a clinical basis. Laboratory procedures were performed to confirm the diagnoses. Gram staining was performed on pus swabs, and cultures were performed on MacConkey and blood agar. The cultures were incubated for 24–48 h for bacterial growth. Specimens were obtained from burrows of suspected scabies lesions for direct microscopy of mites. Skin biopsies were taken as appropriate for routine histological examination. No specific tests were performed to confirm viral lesions.

Hairs, nail clippings, and skin scrapings were obtained as appropriate, treated with 1–2 drops of 20% KOH for 15–30 min, and examined under a microscope for the presence of fungal hyphae. Fungal cultures were performed on Sabouraud’s Dextrose Agar (SDA) for 14 or 28 days. Colonies grown on SDA were transferred to 1.5 mL e-tubes and centrifuged (Eppendorf 5804/R) at 16,260× *g* for 10 min. The colonies were then homogenized with a plastic pestle, the supernatant was collected, and 200 μL of lysis buffer (10 mM EDTA, 100 mM, 1 M KCl, and Tris-HCl (pH 9.5) was added. An amount of 50 ng of proteinase K was added, and the mixture was heated for 16 h at 55 °C followed by heating at 100 °C for 30 min. The mixture was thoroughly shaken. An amount of 400 µL of PCI (phenol: chloroform: isoamyl alcohol; 25:24:1 *v*/*v*) was added and centrifuged at 4 °C for 15 min at 19,083× *g*. DNA was concentrated at −80 °C for an hour after removing the supernatant, and an equivalent volume of isopropanol was added. The purified DNA products were centrifuged at 19,083× *g* for five minutes after being washed twice in 500 µL of 70% ethanol. The DNA was extracted from the supernatant, dried for 20 min at 37 °C, dissolved in distilled water, and stored at 20 °C. A total of 50 ng of the extracted DNA was taken for use in PCR after the concentration was determined using a NanoDrop spectrophotometer. The PsT sets of primers used for fungal identification are summarized in Table 1. The DNA samples were amplified in a reaction mixture with a final volume of 12.5 μL having 0.5 μL of genomic DNA, 1.25 μL of dNTPs (dATP, dGTP, dCTP, dTTP, 2 mM each), 1.25 μL of 10X buffer, 1.5 μL of each primer and 0.25 μL of Taq DNA polymerase (2.5 U/μL). The PCR conditions were as follows: initial denaturation at 96 °C for 2 min, then 30 cycles of denaturation at 96 °C for 30 s, annealing at 63 °C for 3 s, and extension at 74 °C for 120 s [18].

Excel 2016 software (Version 2301) was used to process the findings initially and verify them to ensure the precision of entries [19]. Analysis was performed using the GraphPad Prism version 9 [20]. Data are presented in percentages and a 95% confidence interval (95% CI). A chi-squared test was performed to determine the difference among the proportions of the variables. A *p*-value of less than 0.05 was considered statistically significant.

## 3. Results

The data were gathered from 582 patients who qualified for the inclusion criteria. The mean age of the study participants was 5.7 ± 4.1 years. The youngest patient was aged ten days, whereas the eldest patient was 17 years old. Amongst them, 296 (50.9%) were males, and 286 (49.1%) were females. Most patients in the study were from the province of Punjab (81.6%), a densely populated region of Pakistan. Patients from other regions included Azad Jammu and Kashmir (14.4%), Islamabad (3.3%), and Khyber Pakhtunkhwa (0.7%).

Scabies was the most reported diagnosis among skin conditions. Poor hygiene, low socioeconomic status, illiteracy, and crowded places are the most common reasons for high scabies prevalence in Pakistan [21]. A total of 281 (45.55%) patients were diagnosed with scabies, followed by 114 (19.6%) with eczema, 60 (10.3%) with dermatitis, 33 (5.7%) with T. capitis, 17 (2.9%) with tinea corporis (T. corporis), 16 (2.7%) with impetigo, and 15 (2.6%) with folliculitis. Other conditions included urticaria, burns, infections, head lice infection, tinea inguinalis (T. inguinalis), T. faciei, nappy rashes, alopecia, warts, tinea incognito (T. incognito), tinea cruris (T. cruris), and acne vulgaris. On analysis with the chi-squared test (Table 2), it was observed that adolescents (12–17 years) were most affected by tinea corporis (*p* = 0.015) and acne (*p* = 0.004). However, eczema (*p* = 0.000), dermatitis (*p* = 0.014), and impetigo (*p* = 0.034) were more prevalent among infants and toddlers (0–4 years). Children (5–11 years) had the most head lice infection (*p* = 0.035). Scabies was significantly higher among males than females (*p* = 0.012). Eczema was most prevalent among patients residing in Punjab and Khyber Pakhtunkhwa (*p* = 0.030).

**Figure 1:**
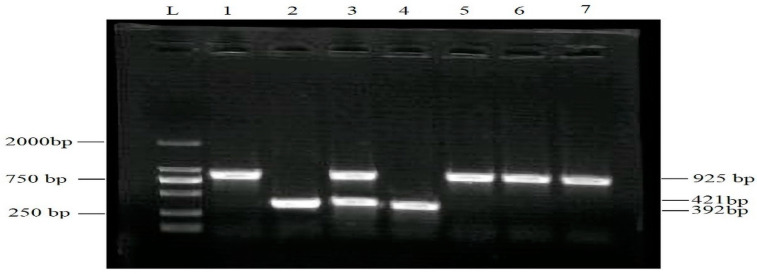
The genomic DNAs of the dermatophyte species were amplified with PCR using PsT primers. L indicates the lane containing DNA marker, lanes 1, 5, 6, 7 indicate specific bands for *T. rubrum* (925 bp), lanes 2 and 4 indicate specific bands for *T. mentagrophytes* (392 bp), and lane 3 shows specific and common *T. violaceum* (421).

PCR successfully amplified a DNA fragment of 925 bp from *T. rubrum*, two DNA fragments of 421 and 925 bp from *T. violaceum*, and a DNA band of 392 bp from *T. mentagrophytes*. Figure 1 shows the gel electrophoresis patterns of the PCR-amplified DNA of various fungal species identified in the present study. The fungal species identified through PCR in T. capitis were *T. violaceum* (52%) and *T. mentagrophytes* (48%), while in T. corporis infestations were *T. rubrum* (39%) and *T. mentagrophytes* (61%).

**Figure 2:**
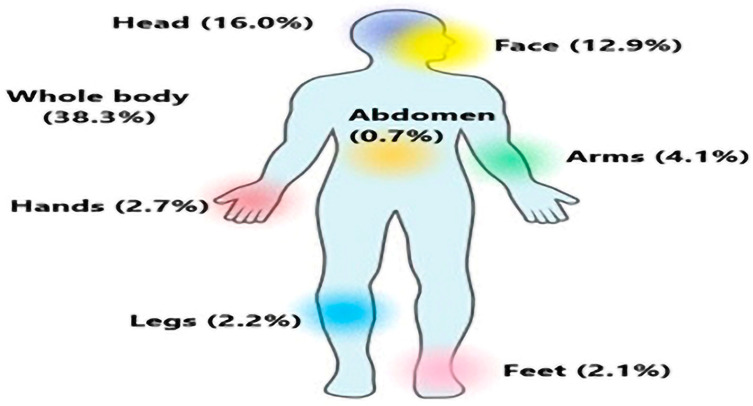
The site-wise percentage distribution of dermatological infections in the body of individuals.

Furthermore, we evaluated which body parts were most affected by an infection. A total of 233 (38.3%) patients were suffering from advanced dermatological conditions. Among the patients, 93 (16.0%) had most infections on their head, followed by 75 (12.9%) on their faces, 24 (4.1%) on their arms, 16 (2.7%) on their hands, 13 (2.2%) on their legs, 12 (2.1%) on their feet, and 4 (0.7%) on their bellies. Females had a significantly higher number of infections in the head than males (*p* = 0.003). On the other hand, more males had their whole bodies affected by skin conditions than females (*p* = 0.021). Similarly, the face and whole body were the most affected areas among infants and toddlers (*p* = 0.009 and *p* = 0.014, respectively), as shown in Figure 2.

The duration of a particular disease was calculated in days, according to the onset of symptoms and consultation. The average duration prior to consultation was 104 ± 209 (0–2190) days.

We also evaluated whether the presence of pets or livestock is a cause of skin infections, with notable cases related to scabies and tinea capitis. Scabies is highly reported in domestic pets, and it can survive in animals for up to 10 years if not treated in time [22]. Children mostly get infected by tinea capitis due to direct contact with pet rodents or infectious outbreaks in the family [23]. It has been reported that zigzag hair is a distinct sign of pet-related T. capitis in neonates caused by domestic animals [24]. Furthermore, it was also observed that birds were the most kept pets. A total of 32 (5.5%) patients reported that they had contact with birds. Another 15 (2.6%) patients were in contact with dogs. Moreover, only 12 (2.1%) patients had contact with livestock, and 6 (1.0%) patients had contact with cats. However, 85.7% of the total sample population had no contact with pets or livestock (Table 3).

This study further determined the number of family members or close relatives affected by skin conditions. It was found that, on average, three family members of a patient were also affected by a skin condition.

We also determined the hygiene habits of the sample population. The present study observed that 60 (10.3%) patients had shared towels or clothes with other family members. Furthermore, 24 (4.1%) also had irregular showering habits. Further analysis showed that scabies was significantly higher among patients with a towel or clothe-sharing practice (*p* = 0.000). Similarly, scabies were also significantly prevalent among patients with once-a-week showers (*p* = 0.000).

## 4. Discussion

The present study highlighted the most common dermatological infections, their causes, and disease duration among children in Punjab, Azad Jammu, and Kashmir, Pakistan. It was found that the most common infestations were scabies and fungal infections. Scabies is a contagious disease caused by skin mites. Its transmission source commonly includes contact with infected people, poor hygiene, and people living in poor economic conditions or health disparities [25]. According to the results of our study, people who contacted scabies mostly came across the disease by contacting a scabies-infected patient or being in unhygienic conditions. Likewise, another case study supporting our findings was that of male soldiers in Pakistan contacting scabies due to certain risk factors that facilitate the infestation of scabies such as unhygienic living conditions, i.e., training outdoors, infrequent bathing, sharing of clothes and towels, and crowded living spaces [26]. Researchers in 2018 reported that scabies account for 38.15% of hospital-based cases, with a high prevalence among male children (53.8%) compared to female children (24%). The highest cases were noted during winter (63.75%) compared to summer (16.89%) due to poor hygiene and living in overcrowded areas [27].

Pakistan is a developing country with a low-middle-income economy with health disparities in villages or suburban areas. The lack of healthcare facilities makes treating diseases ineffective and burdensome, especially for children. In another study, researchers evaluated the risk factors of scabies in the district of Haripur, and people living in resource-poor communities had a higher prevalence of the disease than those with access to better healthcare facilities [28]. Scabies and other skin infections more commonly appeared in children, as shown by our results and further supported by a study in Jamshoro, Sindh, where the prevalence of scabies was up to 34% in all age groups under nine years [29,30]. Similar findings concluded that scabies was most commonly observed in young and older children and less frequently found in adults [31]. The transmission of scabies in a rural community-based study in Brazil reported that Sarcoptes scabiei was the highest contributor to the development of scabies in younger patients (15.5% of patients < 15 years old) in the developing areas [21]. In further supporting evidence, a high prevalence of 50% of scabies was observed in Australian Aboriginals due to a lack of healthcare and resource-poor living [32]. A study in Bangladesh reported that children under six years of age living in a community with poor socioeconomic status developed scabies within one year [33].

Our findings report eczema to be the second most prevalent skin disease (19%), and dermatitis the third most common (10%) in the sample population. The prevalence of skin diseases like eczema and dermatitis depends on several reasons. The clinical reasons for the prevalence of eczema include a history of allergy, breastfeeding, asthma, or autoimmune disorders, whereas other risk factors include demographic distribution, ethnicity, and living in an unhygienic environment without access to healthcare facilities [34]. Atopic and contact dermatitis are chronic inflammatory diseases with risk and contributing factors similar to other contagious diseases, i.e., unhygienic living conditions, allergies, sharing of clothing, frequent exposure to allergens or infected patients, low immune system, and dysfunctional epidermal barrier [35]. Another reason for the high prevalence of such diseases in Pakistan is due to varying weather conditions, especially in the harvesting seasons or tropical areas, as an occupational hazard. A study performed on 701 workers in India associated with the fruit-growing industry of Kashmir Valley reported that dermatitis had become a serious health concern in these areas. Recently, another study reported that scabies spread more in winter and humid environments compared to dry weather, as the survival rate of mites is high during humid weather [36]. The severity of diseases depends on the duration of exposure to the causative and contributing factors [37]. A study with a large cohort of 385,853 participants aged 6–7 years from 143 centers in 60 countries reported the global variations of eczema in children and showed that eczema prevailed in India from 0.9 to 22%, the largest among all the countries [38]. The study concluded that eczema is more prevalent in developing countries. In 2009, researchers presented the pattern of skin diseases in patients of a tertiary care hospital in Pakistan [39]. Out of 95,983 patients, 31.07% were diagnosed with eczema, followed by fungal, viral, and bacterial infections in 28.16%, concluding that most patients presented advanced eczema and infectious diseases. Similar results were presented from patients of the dermatology department in a tertiary care hospital in Karachi, where 1733 patients were enrolled (54% females and 46% males) and reported that infections and infestations were the most commonly reported disorders (37.4%), followed by eczema (36%) [40].

Fungal infections were this study’s third most reported disorders (9%), including tinea capitis, tinea corporis, impetigo, and folliculitis. The prevalence of fungal infections in Pakistan varies in different regions and seasons. However, its actual burden is unknown. The high-risk factors include residence in humid and hot areas, diabetes, tuberculosis, and immune disorders [41]. The most prevalent fungal infections are cutaneous infections. A study was conducted in which they identified the clinical and etiological correlation of tinea capitis in Lahore, Pakistan [42]. Of 180 patients, 95% were children up to 12 years of age with tinea capitis infections caused by *T. violaceum*. Our results were supported by a systemic review reporting tinea capitis infection in school-going children in Africa [43]. The results showed that the highest infection of T. capitis was 23%, caused mainly by Trichophyton species. A study in Pakistan’s Khyber Pakhtunkhwa province revealed that tinea corporis was the most common infection, followed by tinea capitis. Tinea corporis was primarily caused by *T. rubrum*, *T. mentagrophytes*, and Epidermophyton floccosum, while tinea capitis was associated with *T. violaceum*, *T. mentagrophytes*, and *M. ferrugineum* [44]. Conversely, studies from Karachi, Pakistan, reported T. capitis as the most prevalent clinical type, followed by T. corporis and T. ungium, respectively [45,46]. Moreover, Hussain et al. [47] reported that Trichophyton spp is the most dominant, followed by Microsporum spp in the Okara district of Punjab province.

Many factors influence the presence and spread of disease symptoms, and a few of them were evaluated in this study, including contact with infected family members and livestock. The participating patients reported being in contact with 2–3 family members previously suffering from the disease, while a few were in contact with birds, cats, or dogs. Scientists reported that dermatophytes (animals causing skin infections) live under the animals’ hair, skin, fur/feathers, and nails, either kept in laboratory settings or domesticated animals [48]. These animals can cause skin diseases such as fungal infections, asthma, or dermatitis. Pet-related infections are most common in our study and are supported by other researchers. In 2013, a study found a positive link between household pets and dermatological infections among children, with most infected individuals being in contact with animals less than a month ago [49]. Several studies report a high prevalence of fungal infections in cows, dogs, and goats [50]. In another study, researchers reported that the most common infections in the USA caused by household pets include scabies or fungal diseases, such as tinea corporis/capitis, along with campylobacter infections or viral-borne diseases such as rabies [51]. Previous studies reported that in canine gums, Alternaria and Cladosporium, two of the most common fungal allergens in human environmental allergies, are present and are most likely to spread these infections to humans as well [52]. Since the participants in our study were in contact with pets, it is plausible that some of their tinea infections were due to animal exposure. Zoonotic infections, such as dermatophytosis (ringworm), resulting from contact with infected cats and dogs or avian mite dermatitis are frequently present as pruritic eruptions or cutaneous lesions [53]. Wounds inflicted by feline bites can lead to secondary bacterial skin infections [54]. Moreover, sensitized children exposed to animal dander may develop conditions like atopic dermatitis or urticaria due to allergenic triggers [55].

## 5. Conclusions

The current study was conducted to evaluate the prevalence of common dermatological conditions among individuals up to the age of 17 years in Punjab, Pakistan. Scabies, eczema, tinea capitis, tinea corporis, impetigo, and folliculitis were the most prevalent skin infections, followed by other conditions like urticaria, burns, infections, head lice infection, tinea inguinalis, tinea faciei, nappy rashes, alopecia, warts, tinea incognito, tinea cruris, and acne vulgaris. Poor hygiene and pets were found to be risk factors for various skin infections. Dermatological conditions are common in younger individuals, but unfortunately, many children do not receive the desired medical assistance.

## 6. Limitations

The present study was limited to the Punjab Province, Islamabad, Azad Jammu and Kashmir, and Khyber Pakhtunkhwa because of the lack of access to the remaining provinces of Pakistan. In addition, this study only includes the reported cases, and there may be many unreported cases. Patients mostly visited when their condition was worse; otherwise, they would not report, so it cannot be considered the actual percentage distribution of skin diseases in Pakistan.

## Figures and Tables

**Table 1:** Set of specific primers used for the detection of dermatophytes, including *Trichophyton rubrum* (*T. rubrum*), *Trichophyton violaceum* (*T. violaceus*), *Trichophyton mentagrophytes* (*T. mentagrophytes*), and Microsporum species. The target amplicon sizes for *T. rubrum*, *T. violaceum*, *T. mentagrophytes*, and Microsporum species were 925 bp, 421 bp, 925 bp, 392 bp, and 522 bp, respectively.

Target Species	Forward Primer	Amplicon Size
*T. rubrum*	dMF2/86: RCGAGGAGAGGACCCRACHTCTGAC dMR2/138: TTCCTTAGTACCRGCYTTG	925 bp
*T. violaceum*	TVCF2/34: GATCCACAAGGTATGTATTAGTTA TVCR2/76: GGTGCCAGCCATGTCGTAGAC	421 bp
*T. mentagrophytes*	TRBF2/253: GCCTGTTGTTCCGCTCATTCTT TRBR2/346: CGGCTAGGAGGGCGTGGTAGAA	392 bp
*Microsporum* spp.	TMTF2/38: GCATGATTTAGAAGTGTAATGCTG MCNR2/138: TTCCTTGGTACCAGCTTTG	522 bp

**Table 2:** Percentage distribution (95% confidence interval) of various dermatological disorders in different age groups (row-wise). The chi-square test revealed a significant (*p* < 0.0001) difference among the proportions of conditions among age stratifications.

Disorders	Age Stratification				
	Infants and Toddlers	Children	Adolescents	Total Percentage	*p*-Value
Scabies	5.55% (0.28–25.75)	33.33% (16.27–56.25)	16.66% (5.83–39.22)	45.55% (41.66–49.49)	<0.0001
Eczema	17.64% (6.19–41.02)	35.29% (17.3–58.69)	17.64% (6.19–41.02)	18.48% (15.62–21.74)	
Dermatitis	21.42% (7.57–47.58)	35.71% (16.34–61.23)	0% (0–21.53)	9.73% (7.63–12.32)	
Tinea capitis	0% (0–24.24)	66.66% (39.06–86.18)	16.66% (2.96–44.8)	5.35% (3.84–7.42)	
Tinea corporis	0% (0–43.44)	100% (56.55–100)	0% (0–43.44)	2.76% (1.73–4.37)	
Impetigo	0% (0–48.98)	50% (8.88–91.11)	50% (8.88–91.11)	2.6% (1.61–4.18)	
Folliculitis	0% (0–56.14)	33.33% (1.7–88.15)	33.33% (1.7–88.15)	2.44% (1.48–3.98)	
Others	0% (0–56.14)	100% (43.85–100)	0% (0–56.14)	13.13% (10.69–16.03)	

**Table 3:** Percentage distribution (95% confidence interval) of different dermatological disorders in association with the patterns of pets (row-wise). The chi-square test did not show a significant (*p* = 0.2402) difference among the proportions of dermatological conditions associated with pet keeping.

Disorders	Dog	Cats	Birds	Livestock	Total	*p*-Value
Scabies	9.63% (4.96–17.88)	1.2% (0.06–6.51)	7.22% (3.35–14.88)	3.61% (0.98–10.09)	22.22% (6.96–17.29)	0.2402
Tinea capitis	6.02% (2.6–13.33)	3.61% (0.98–10.09)	7.22% (3.35–14.88)	3.61% (0.98–10.09)	20.84% (6.42–16.48)	
Dermatitis	7.22% (3.35–14.88)	3.61% (0.98–10.09)	6.02% (2.6–13.33)	0% (0–4.42)	15.28% (4.32–13.17)	
Eczema	2.4% (0.42–8.36)	0% (0–4.42)	9.63% (4.96–17.88)	2.4% (0.42–8.36)	13.89% (3.82–12.32)	
Tinea inguinal	0% (0–4.42)	0% (0–4.42)	6.02% (2.6–13.33)	0% (0–4.42)	5.56% (1.09–6.93)	
Tinea corporis	0% (0–4.42)	0% (0–4.42)	2.4% (0.42–8.36)	2.4% (0.42–8.36)	5.56% (1.09–6.93)	
Urticaria	1.2% (0.06–6.51)	0% (0–4.42)	1.2% (0.06–6.51)	1.2% (0.06–6.51)	4.17% (0.57–5.95)	
Tinea faciei	0% (0–4.42)	0% (0–4.42)	3.61% (0.98–10.09)	0% (0–4.42)	4.17% (0.57–5.95)	
Others	0% (0–4.42)	0% (0–4.42)	7.22% (3.35–14.88)	1.2% (0.06–6.51)	8.33% (1.93–8.8)	

## Data Availability

All the data is available in the manuscript.

## References

[1] Esposito S., Fumagalli M., Principi N. (2012). Immunogenicity, safety and tolerability of vaccinations in premature infants. Expert Rev. Vaccines.

[2] Ghirano I.A., Sheikh S., Arain A.A. (2017). Skin diseases: Prevalence in pediatric patients in Hyderabad: Sindh, Pakistan. Prof. Med. J..

[3] Laube S. (2004). Skin infections and ageing. Ageing Res. Rev..

[4] Carlsten C., Dimich-Ward H., Ferguson A., Watson W., Rousseau R., DyBuncio A., Becker A., Chan-Yeung M. (2013). Atopic dermatitis in a high-risk cohort: Natural history, associated allergic outcomes, and risk factors. Ann. Allergy Asthma Immunol..

[5] Ma L., Chen M., Fa Z., Pan W., Liao W., Gao X.-H., Huo W., Yang Y., Chen H.-D., Holahan H. (2016). Skin diseases caused by factors from the Environment. Practical Immunodermatology.

[6] Chung J., Simpson E.L. (2019). The socioeconomics of atopic dermatitis. Ann. Allergy Asthma Immunol..

[7] Memon K.N., Soomro R.A., Ansari M.S. (2011). Pattern of skin diseases in patients visiting a tertiary care health facility at Hyderabad, Pakistan. J. Ayub Med. Coll. Abbottabad.

[8] Banerji A. (2015). Scabies. Paediatr. Child Health.

[9] Rathi S., Rathi H., Lakhani H., Hansotia M. (2001). Awareness about scabies among general medical practitioners (GPs) of Karachi, Pakistan. J. Pak. Med. Assoc..

[10] Brown G.D., Denning D.W., Gow N.A., Levitz S.M., Netea M.G., White T.C. (2012). Hidden killers: Human fungal infections. Sci. Transl. Med..

[11] Jain A., Jain S., Rawat S. (2010). Emerging fungal infections among children: A review on its clinical manifestations, diagnosis, and prevention. J. Pharm. Bioallied Sci..

[12] Hoare C., Li Wan Po A., Williams H. (2001). Systematic review of treatments for atopic eczema. Health Technol. Assess..

[13] Sandhu J.K., Salame N., Ehsani-Chimeh N., Armstrong A.W. (2019). Economic burden of cutaneous infections in children and adults with atopic dermatitis. Pediatr. Dermatol..

[14] Coe A. (2016). Eczema—An Itchy Problem. Don’t Forget the Bubbles. https://dontforgetthebubbles.com.

[15] Myers J.M.B., Hershey G.K.K. (2010). Eczema in early life: Genetics, the skin barrier, and lessons learned from birth cohort studies. J. Pediatr..

[16] Nuzhat Y., Mohammad Riaz K. (2005). Spectrum of common childhood skin disease: A single centre experience. J. Pak. Med. Assoc..

[17] Thrusfield M. (2007). What sample size should be selected. Veterinary Epidemiology.

[18] Kanbe T., Suzuki Y., Kamiya A., Mochizuki T., Fujihiro M., Kikuchi A. (2003). PCR-based identification of common dermatophyte species using primer sets specific for the DNA topoisomerase II genes. J. Dermatol. Sci..

[19] Held B., Moriarty B., Richardson T. (2019). Microsoft Excel Functions and Formulas with Excel 2019/Office 365.

[20] Mavrevski R., Traykov M., Trenchev I., Trencheva M. (2018). Approaches to modeling of biological experimental data with GraphPad Prism software. WSEAS Trans. Syst. Control.

[21] Zeba N., Shaikh D.M., Memon K.N., Khoharo H.K. (2014). Scabies in relation to hygiene and other factors in patients visiting Liaquat University Hospital, Sindh, Pakistan. Age Years.

[22] Kandi V. (2017). Laboratory diagnosis of scabies using a simple saline mount: A clinical microbiologist’s report. Cureus.

[23] Nenoff P., Krüger C., Ginter-Hanselmayer G., Tietz H.J. (2014). Mycology–an update. Part 1: Dermatomycoses: Causative agents, epidemiology and pathogenesis. JDDG J. Dtsch. Dermatol. Ges..

[24] Zhi H.-L., Xia X.-J., Shen H., Lv W.-W., Zhong Y., Sang B., Li Q.-P., Liu Z.-H. (2023). Trichoscopy for early diagnosis and follow-up of pet-related neonatal tinea capitis. Mycopathologia.

[25] Ahmadi B., Mirhendi H., Shidfar M., Nouripour-Sisakht S., Jalalizand N., Geramishoar M., Shokoohi G. (2015). A comparative study on morphological versus molecular identification of dermatophyte isolates. J. Mycol. Méd..

[26] Thadchanamoorthy V., Dayasiri K. (2020). Diagnosis and management of scabies in children. Sri Lanka J. Child Health.

[27] Rizvi A., Rossi L. (2018). Scabies prevalence and risk factors in Pakistan: A hospital based survey. Biomed. J..

[28] Raza N., Qadir S., Agha H. (2009). Risk factors for scabies among male soldiers in Pakistan: Case-control study. EMHJ-East. Mediterr. Health J..

[29] Khatoon N., Khan A., Azmi M.A., Khan A., Shaukat S.S. (2016). Most common body parts infected with scabies in children and its control. Pak. J. Pharm. Sci..

[30] Yasmin S. (2016). Epidemiological study of scabies in district Haripur, Pakistan. Arthropods.

[31] Hussain Bux A.K., Abdul Sattar C., Aijaz Hussain M. (2012). Scabies in community of Jamshoro hills. Med. Forum Mon..

[32] Jackson A., Heukelbach J., Feldmeier H. (2007). Transmission of scabies in a rural community. Braz. J. Infect. Dis..

[33] Currie B.J., Carapetis J.R. (2000). Skin infections and infestations in Aboriginal communities in northern Australia. Australas. J. Dermatol..

[34] Stanton B., Khanam S., Nazrul H., Nurani S., Khair T. (1987). Scabies in urban Bangladesh. J. Trop. Med. Hyg..

[35] Martin P., Koplin J., Eckert J., Lowe A., Ponsonby A.L., Osborne N., Gurrin L., Robinson M., Hill D., Tang M. (2013). The prevalence and socio-demographic risk factors of clinical eczema in infancy: A population-based observational study. Clin. Exp. Allergy.

[36] Browne E., Driessen M.M., Ross R., Roach M., Carver S. (2021). Environmental suitability of bare-nosed wombat burrows for Sarcoptes scabiei. Int. J. Parasitol. Parasites Wildl..

[37] Nutten S. (2015). Atopic dermatitis: Global epidemiology and risk factors. Ann. Nutr. Metab..

[38] Bashir Y., Hassan I., Zeerak S., Bhat M.A., Jeelani S., Bhat Y.J., Rather S.P., Bashir S. (2022). Profile of Dermatological Disorders Among Workers Involved in Fruit Growing Industry of Kashmir Valley in North India. Indian Dermatol. Online J..

[39] Odhiambo J.A., Williams H.C., Clayton T.O., Robertson C.F., Asher M.I., Group I.P.T.S. (2009). Global variations in prevalence of eczema symptoms in children from ISAAC Phase Three. J. Allergy Clin. Immunol..

[40] Aman S., Nadeem M., Mahmood K., Ghafoor M.B. (2017). Pattern of skin diseases among patients attending a tertiary care hospital in Lahore, Pakistan. J. Taibah Univ. Med. Sci..

[41] Maryum H., Alam M.Z., Ahmed I. (2014). Pattern of skin diseases in a tertiary care private hospital, Karachi. J. Pak. Assoc. Dermatol..

[42] Jabeen K., Farooqi J., Mirza S., Denning D., Zafar A. (2017). Serious fungal infections in Pakistan. Eur. J. Clin. Microbiol. Infect. Dis..

[43] Hussain I., Aman S., Haroon T., Jahangir M., Nagi A. (1994). Tinea capitis in Lahore, Pakistan. Int. J. Dermatol..

[44] Usman B., Rehman A., Naz I., Anees M. (2021). Prevalence and antifungal drug resistance of dermatophytes in the clinical samples from Pakistan. Acta Microbiol. Immunol. Hung..

[45] Kashif S., Uddin F., Nasir F., Zafar S., Jabeen S., Kumar S. (2021). Prevalence of dermatophytes in superficial skin infections in a tertiary care hospital. J. Pak. Assoc. Dermatol..

[46] Ansari F., Siddiqui S.A. (2006). Prevalence of dermatophytic infections in Karachi, Pakistan during the year 2003–2004. Pak. J. Bot..

[47] Hussain A., Zakki S.A., Qureshi R. (2016). Epidemiological Study of Dermatophytosis in Okara, Pakistan. RADS J. Pharm. Pharm. Sci..

[48] Bongomin F., Olum R., Nsenga L., Namusobya M., Russell L., de Sousa E., Osaigbovo I.I., Kwizera R., Baluku J.B. (2021). Estimation of the burden of tinea capitis among children in Africa. Mycoses.

[49] Jehangir F., Vohra E.A. (2013). Frequency of Tinea Capitis in Children 5–15 Years of Age Presenting to Primary Health Care Centre in Karachi, Pakistan. Infect. Dis. J. Pak..

[50] Hameed K., Ch F.R., Nawaz M.A., Naqvi S.M.S., Gräser Y., Kupsch C., Pasquetti M., Rossi L., Min A.R.M., Tizzani P. (2017). Trichophyton verrucosum infection in livestock in the Chitral district of Pakistan. J. Infect. Dev. Ctries..

[51] Connole M., Yamaguchi H., Elad D., Hasegawa A., Segal E., Torres-Rodriguez J. (2000). Natural pathogens of laboratory animals and their effects on research. Med. Mycol..

[52] Rabinowitz P.M., Gordon Z., Odofin L. (2007). Pet-related infections. Am. Fam. Physician.

[53] Chomel B.B. (2016). Diseases transmitted by less common house pets. Infections of Leisure.

[54] Oehler R.L., Velez A.P., Mizrachi M., Lamarche J., Gompf S. (2009). Bite-related and septic syndromes caused by cats and dogs. Lancet Infect. Dis..

[55] Jenerowicz D., Silny W., Danczak-Pazdrowska A., Polanska A., Osmola-Mankowska A., Olek-Hrab K. (2012). Environmental factors and allergic diseases. Ann. Agric. Environ. Med..

